# The Role of Back Muscle Dysfunctions in Chronic Low Back Pain: State-of-the-Art and Clinical Implications

**DOI:** 10.3390/jcm12175510

**Published:** 2023-08-24

**Authors:** Thomas Matheve, Paul Hodges, Lieven Danneels

**Affiliations:** 1 Spine, Head and Pain Research Unit Ghent, Department of Rehabilitation Sciences, Ghent University, 9000 Gent, Belgium; lieven.danneels@ugent.be; 2 REVAL—Rehabilitation Research Center, Faculty of Rehabilitation Sciences, UHasselt, 3500 Diepenbeek, Belgium; 3 NHMRC—Centre of Clinical Research Excellence in Spinal Pain, Injury & Health, School of Health & Rehabilitation Sciences, The University of Queensland, Brisbane 4072, Australia; p.hodges@uq.edu.au

**Keywords:** back pain, muscle dysfunction, clinical implications

## Abstract

Changes in back muscle function and structure are highly prevalent in patients with chronic low back pain (CLBP). Since large heterogeneity in clinical presentation and back muscle dysfunctions exists within this population, the potential role of back muscle dysfunctions in the persistence of low back pain differs between individuals. Consequently, interventions should be tailored to the individual patient and be based on a thorough clinical examination taking into account the multidimensional nature of CLBP. Considering the complexity of this process, we will provide a state-of-the-art update on back muscle dysfunctions in patients with CLBP and their implications for treatment. To this end, we will first give an overview of (1) dysfunctions in back muscle structure and function, (2) the potential of exercise therapy to address these dysfunctions, and (3) the relationship between changes in back muscle dysfunctions and clinical parameters. In a second part, we will describe a framework for an individualised approach for back muscle training in patients with CLBP.

## 1. Introduction

Low back pain (LBP) is one of the leading causes of disability worldwide and has an enormous impact on a personal and societal level [1,2]. About 80% of the population will experience an episode of LBP during their lifetime [3]. Although an episode of acute LBP usually resolves within a few weeks, up to two thirds of patients report a flare-up within one year and about 15% will develop chronic low back pain (CLBP) [4,5,6,7], which is typically defined as LBP lasting for more than three months. The multidimensional nature of CLBP has been widely accepted [8,9]. Acknowledging the relative contribution of different factors to CLBP—including physical, emotional, cognitive, lifestyle, social, and behavioural aspects—is essential, as they will guide the assessment and treatment of the individual patient [10,11].

**Figure 1:**
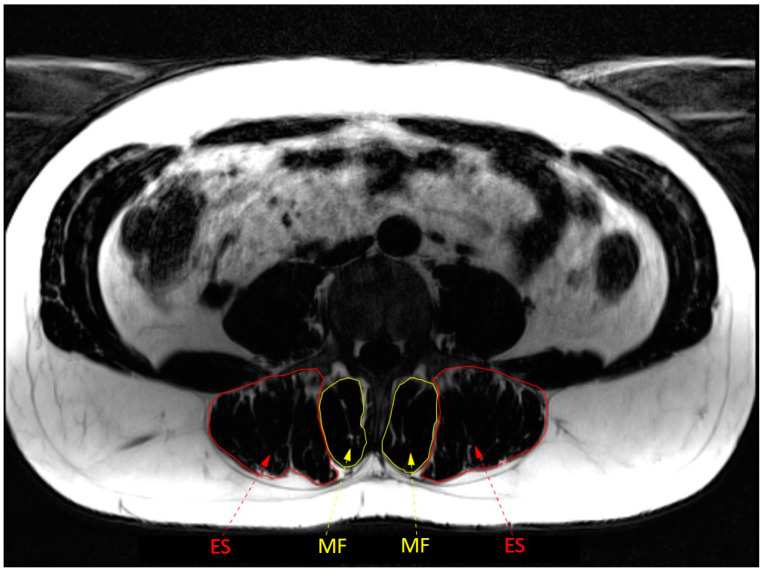
Anatomy of back muscles at L4 level. ES = erector spinae; MF = multifidus.

Two important physical factors are the structure and function of the back muscles, in particular of the lumbar multifidus and erector spinae (see Figure 1) [12,13]. The lumbar multifidus is the most medial back muscle in the lumbar region [13]. The multifidus muscle includes short deep fibres that span two intervertebral segments (referred to as the deep multifidus), and more superficially located muscle fibres that span three to five vertebral segments (referred to as superficial multifidus) [13]. The erector spinae is located laterally to the lumbar multifidus and consists of the lumbar and thoracic portions of the longissimus and iliocostalis muscles [13]. Due to its location and anatomy, the deep multifidus has little potential to extend the lumbar spine and mainly provides compressive forces that are important for segmental control [14]. Because of their more superficial location and longer lever arms, the superficial multifidus and erector spinae have a greater contribution to lumbar spine extension [13]. When they contract asymmetrically, they also contribute to sidebending and rotation [13,14].

Evidence is emerging that changes in back muscle function and structure are time-dependent and exist on a continuum from acute to chronic LBP [12,15]. One potential implication of this is that different treatments are likely to be required to target these features depending on the timepoint on this trajectory towards chronicity. Of note, even within subgroups based on the time-course—and especially in patients with CLBP—there is large variability in the features of back muscle structure and function, and their role in the persistence of back problems is likely to differ between individuals [10]. This implies that interventions should always be based on a thorough examination taking into account the specific presentation of back muscle changes and the other multidimensional features of CLBP. This can be complex.

The objectives of this paper are to provide a state-of-the-art update of features of back muscle structure and function in patients with CLBP and their potential implications for treatment. To this end, the paper first gives an overview of (1) dysfunctions in back muscle structure and function, (2) the potential for exercise therapy to address these dysfunctions, and (3) the relationship between changes in back muscle dysfunctions and clinical parameters. In a second part, a framework is described for an individualised approach for back muscle training patients with CLBP.

## 2. Back Muscle Dysfunctions in Patients with Chronic Low Back Pain

### 2.1. Methods

To ensure the inclusion of the most recent and relevant information in this state-of-the-art overview of back muscle dysfunctions in CLBP, we conducted literature searches in the Pubmed and Web of Science databases up until April 2023. Search terms were partly derived from earlier conducted (systematic) reviews on the topics included in this overview (e.g., [16,17,18,19]). We selected papers including adults with CLBP that contained relevant information consistent with our three main objectives of this overview, i.e., to describe (1) dysfunctions in back muscle structure and function, (2) the potential for exercise therapy to address these dysfunctions, and (3) the relationship between changes in back muscle dysfunctions and clinical parameters. Both original research and literature reviews were considered. 

### 2.2. Muscle Structure

Persistence of LBP is associated with extensive changes in the structure of the back muscles. Several studies have identified bilateral reduction in the multifidus cross-sectional area (CSA) and sometimes over several spinal levels in CLBP [20,21,22,23,24]. This differs from the more localized reduction of CSA (which can be specific to the painful side in unilateral conditions) in acute LBP. Findings for other muscles vary across studies [24]. Some studies report atrophy of the combined erector spinae and multifidus [25], whereas others report atrophy of the multifidus alone in CLBP [20,21]. Smaller CSAs of the multifidus, psoas, and quadratus lumborum muscle have been reported in some cases of LBP of longer duration [26]. Some studies comparing measures between individuals with continuous LBP and intermittent LBP in remission found no differences in the multifidus or erector spinae CSA [15,27].

Fatty infiltrations, either restricted to the multifidus or more widespread, have been shown using both qualitative [24,25,28] and quantitative [27] methods. Overall, patients with CLBP have a greater CSA of fat in the multifidus and to a lesser extent in erector spinae when compared to pain-free persons [24]. Moreover, the fat CSA and lean muscle fat index (indicating more fatty infiltration) are greater in the multifidus and erector spinae in cases of continuous CLBP (i.e., 7 pain days/week) than in individuals with recurrent LBP and noncontinuous CLBP (i.e., 3–4 pain days/week) [15]. Computed tomography measures have not found generalized fatty infiltration across the back muscles [21], but muscle density measures using computed tomography (which might be related to differences in fat content) show lower values in the multifidus and erector spinae at levels with facet joint osteoarthritis, spondylolisthesis, and intervertebral disc narrowing [29]. Experimental animal studies have shown a progression from localized to multisegmental changes in muscle structure over time after injury to a single intervertebral disc [30].

Findings regarding muscle-fibre-type proportions in CLBP are variable [14,16,17]. One study showed lower proportions of type I fibres and higher proportions of type II fibres in patients with CLBP scheduled for spinal surgery [31]. This study found no difference in the CSA of individual fibres, suggesting a smaller area occupied by type I fibres [31]. Another study in patients with CLBP scheduled for surgery also reported a higher proportion of type II fibres compared to pain-free persons, but found a smaller CSA of both Type I and Type II fibres in the CLBP group [32]. These results were independent of physical activity levels in the CLBP group [32]. A negative correlation between the proportion of type I fibres and the duration of pain, but a positive correlation with type IIx fibres, has also been observed [33]. T2 resting values also suggest a tendency towards a higher proportion of type II fibres in the multifidus and erector spinae in LBP [15]. Not all studies support these observations. Some studies found no differences in fibre size [34] or type I fibre proportion [34,35] in mild disabling LBP, despite poorer performance on a back muscle endurance test [34]. Variations in findings may be explained by symptom severity or the presence of spinal pathology. For instance, greater fibre II type proportions compared to pain-free persons have been reported for individuals undergoing surgery [31,32], whereas some studies in mild LBP found no differences [34,35]. Other aspects, such as variations in biopsy locations and the harvesting of control samples from cadavers with unclear LBP history may also contribute to variation in findings. Moreover, different methods have been used to classify muscle fibre types, such as myosin ATPase histochemical staining [31,33,34], expression of myosin heavy chain isoforms [35] or other methods [15,32]. This affects reported variations in type II classification [17], and some studies also do not specify the type II subclassification (e.g., IIa vs. IIx/d [36]) [15,32,34]. These issues complicate the interpretation of results and comparisons between studies. Finally, it is important to consider that all human studies are cross-sectional, and no longitudinal data are available. Longitudinal human studies are warranted to provide more insight into these aspects [12].

The mechanisms underlying structural muscle changes in CLBP are not completely understood, but are thought to differ over the time-course of the condition. In acute back pain, neurologically mediated reflex inhibition has been speculated in humans [37] and supported by animal data [38]. In the subacute period, there is emerging evidence from animal studies for changes mediated by the immune system [30] that have been supported by human data [39]. In the chronic phase, the features of muscle structure might be explained by deconditioning [12]. Reduced capacity due to earlier neural and inflammatory mechanisms may transition to reduced function [40,41]. Conditions that compromise the intervertebral foramen, such as spinal stenosis [42] and intervertebral disc disease [40], might lead to muscle atrophy and fat infiltration via mechanisms of denervation.

In sum, extensive changes in back muscle structure are present in patients with CLBP. In particular, the lumbar multifidus has a smaller bilateral CSA and increased fatty infiltration. These changes are less clear or less pronounced for other muscles. Findings regarding muscle-fibre-type proportions in CLBP are variable. When interpreting these results, it is important to consider the heterogeneity in the CLBP population as changes in muscle structure seem to be more pronounced in patients with more severe complaints (e.g., more continuous and/or more disabling LBP). See Table 1 for a summary of changes in back muscle structure in patients with CLBP. 

### 2.3. Back Muscle Function

There is a large body of literature that has evaluated and reviewed features of sensorimotor control of the back muscles that differ between individuals with and without back pain. This section considers some specific features, including recent observations, that have relevance for designing interventions. For comprehensive reviews see [12,43].

#### 2.3.1. Sensorimotor Control

Back muscles make an important contribution to the control of spinal posture. They are activated in advance of perturbations that are predictable and react with short latency to perturbations that are not predictable. A recent systematic review concluded that differences in the reaction times of erector spinae to predictable and unpredictable perturbations were variable between individuals with and without low back pain—some studies show delayed reaction times and other studies found no differences [18]. There are many potential explanations for the variation including differences in recording electrodes, different task, back pain patients with different presentations, and different methods to quantify the timing of muscle activation. In the studies that do report differences in erector spinae reaction time, they are typically delayed [18,19]. Although these data appear to suggest some compromise in the activation of back muscles (which implies suboptimal control) [12], there are also data that suggest excessive recruitment of back muscles in response to experimental pain [44] and in individuals with chronic back pain during functional tasks [12,45], especially in those with unhelpful beliefs [46,47]. Increased activation appears to more consistently involve the more superficial erector spinae than deep (e.g., multifidus) muscles [12].

Other work has examined the sensorimotor mechanisms for control of back muscles by evaluation of the response of the muscles to transcranial magnetic stimulation over the motor cortex. Some studies have revealed reduced excitability of the descending pathways to the erector spinae [48] and alterations in the motor cortex representation of the back muscles [49]. Notably, this altered representation was characterized by the merging of distinct brain representations of the deep multifidus and superficial erector spinae muscles. This phenomenon has been found to correlate with the severity of LBP [50], particularly in individuals who have poorer capacity to differentiate between lumbar and thoracolumbar motion [51]. Changes in corticomotor function provide support for compromised multifidus muscle function in LBP. However, further research is necessary to fully comprehend the relationship between brain changes, motor function, and symptoms associated with LBP [12].

Studies that have investigated the somatosensory system in LBP have identified less disturbance to postural control from stimulation of proprioceptive signals from the back muscles in standing positions [52,53,54], which might indicate that information on back position/movement is weighted down. Patients with LBP also have impaired lumbar proprioception compared with controls when measured actively in sitting positions (especially when patients are categorised in direction-specific subgroups) or via a threshold to the detection of passive motion [55,56].

In conclusion, timing of erector spinae activation to predictable and unpredictable perturbations varies between patients with CLBP, but if impairments are present, they are characterized by delayed activation. Increased activity of erector spinae (as opposed to lumbar multifidus) during functional tasks is often observed in individuals with CLBP, which may represent a protective movement strategy. Motor cortex changes in areas representing the back muscles are related to compromised multifidus muscle function in LBP. Conversely, patients with LBP typically reduce the weighting of afferent proprioceptive information from the back muscles (mainly multifidus) for maintaining postural control. This indicates that changes in both ‘top down’ and ‘bottom up’ mechanisms are involved in sensorimotor control impairments in LBP. See Table 2 for a summary of changes in selected back muscle functions in patients with CLBP.

#### 2.3.2. Spatial Distribution of Lumbar Back Muscle Activity

Activity of superficial muscles is often assessed using bipolar surface electromyography, which limits the evaluation of muscle activity to a few separate lumbar areas. High-density surface electromyography can overcome this limitation, as this method uses a grid of multiple small electrodes (e.g., 5 × 13 electrodes) with small inter-electrode distance (e.g., 8 mm). Typically, the bottom end of this grid is placed 2 cm lateral to the L5 spinous process, covering the lumbar erector spinae up to approximately L2 [57,58,59]. This allows the measurement of the spatial distribution (i.e., which areas of the erector spinae are active) and spatiotemporal changes in superficial muscle activity during repeated or sustained tasks with more detail than traditional bipolar surface electromyography [58,60]. Some recent work with high-density surface electromyography has provided new insight into spatial distribution and spatiotemporal changes in erector spinae in patients with CLBP.

Alterations in spatial distribution of erector spinae muscle activity have been observed in CLBP, but differences appear to be task-dependent. During tasks that induce higher levels of muscle activity and muscle fatigue, such as repeated lifting or muscle endurance tests, individuals with CLBP typically use more cranially located regions of the erector spinae compared to pain-free persons [61,62,63,64]. Moreover, those with CLBP also have less dispersed erector spinae muscle activity during these type of tasks [63,64,65]. These differences relative to pain-free individuals have not been observed for low-load activities, such as walking or sit-to-stand [66]. The importance of muscle activity levels is shown by Arvanitidis et al. [62], who used a 15 s isometric back extension exercise at 20% and 50% of erector spinae MVC. During the low-load task, both patients with CLBP and pain-free persons used an equally dispersed activation pattern of erector spinae. During the high-load task, erector spinae activity was located more cranially in patients with CLBP, while the opposite pattern was observed in the pain-free persons.

In pain-free persons, spatiotemporal changes in erector spinae activity are typically present during repetitive or prolonged fatiguing tasks [59,63,65,67]. However, this redistribution of lumbar erector spinae activity does not seem to follow a stereotypical pattern, as both caudal and cranial shifts in erector spinae activity have been observed [59,63,67]. This suggests that motor control strategies to redistribute muscle activity might be specific to the individual and task. It is currently unclear whether there are differences in redistribution in erector spinae activity in patients with CLBP relative to that observed in pain-free persons, because some studies have reported impairments [59,65] whereas others have not [61,68]. In studies that have reported impairments in spatial (re)distribution of erector spinae in CLBP, these impairments have been related to increased pain during [59,65] and poorer performance [63] on repetitive or endurance tasks. This failure to redistribute with fatigue appears consistent with the hypothesis that variation in muscle activation acts to reduce fatigue and prevent tissue overloading, thus protecting against the development of pain [69]. The absence of impairments of (re)distribution of erector spinae activity in 25–35% of patients with CLBP [59,63] might explain why some studies do not find between-group differences.

Both central and peripheral mechanisms have been put forward to explain impaired redistribution of back muscle activity [70]. Motor adaptations to acute pain that are driven by the nervous system are thought to protect body tissues from potential or actual injury [71]. Although it is not exactly clear why these adaptations may persist when protection is no longer necessary in the absence of nociceptive pain, pain-related psychological factors may play a role in this process [71,72]. Preliminary evidence supports a potential relationship with psychological features—spatial redistribution of erector spinae is less in patients with acute LBP [73] and was decreased during a repetitive lifting task in pain-free persons who perceived this task as more harmful [57]. Alternatively, redistribution of muscle activity may be hampered in patients with structural changes in the back muscles, such as increased fatty infiltration, fibrosis or fast-twitch muscle fibres [70]. These changes in muscle quality would be expected to increase metabolic demand and accelerate fatigue [15], and as these changes are more profound in caudal lumbar regions this might underlie the more cranial and less distributed activity.

In summary, individuals with CLBP appear to activate different and less diffuse areas of the back muscles during fatiguing tasks compared to pain-free controls. As impairments in spatial (re)distribution of erector spinae activity have been associated with pain and fatigue, these features could possibly be a potential treatment target [60]. 

#### 2.3.3. Back Muscle Strength and Endurance

In their review, Steele et al. (2014) concluded that patients with (chronic) LBP have decreased lumbar extensor strength and endurance [74]. This has been confirmed by the recent literature [63,75,76,77,78,79,80,81,82,83,84,85,86,87]. However, this is not supported by all studies and the effect sizes of differences between individuals with and without CLBP are variable [86,87,88]. Although this may be partly due to variations in assessment protocols, such as differences in participant positioning (e.g., sit vs. stance) or type of exercise (e.g., isometric vs. isokinetic), this variability in between-group effect sizes is also observed when the same testing protocols are used. For example, small to very large effect sizes in differences in back muscle endurance have been reported between patients with CLBP and pain-free persons when measured with the Biering–Sorensen test [87,89]. Large variability is also present between results from studies using the same (or very similar) testing protocols in the same population. For example, time to failure during the Biering–Sorensen test in pain-free persons has been reported to range between 78 and 221 s [90,91], while in patients with CLBP, time to failure ranges between 39 and 144 s [84,90]. This variation is not unexpected as it would be naïve to assume that all patients with this highly heterogeneous condition would present in a similar manner. Many features can account for the variation. 

Besides demographic (e.g., age or sex) and anthropometric (e.g., BMI) variables, it has frequently been suggested that pain-related psychological factors may substantially contribute to the variability in muscle strength and endurance in patients with CLBP [92]. For example, patients with higher levels of fear of movement may terminate the test prematurely; in a simple manner, this might relate to a belief that the task might cause pain or injury. Contrary to this hypothesis, a recent meta-analysis only found very small associations between pain-related psychological factors and muscle strength and endurance tests in patients with CLBP [93]. However, this might not tell the whole story—pain-related psychological factors are typically assessed using generic self-report measures, such as the Tampa Scale for Kinesiophobia [94,95] and these generic measures do not capture a patient’s beliefs regarding specific activities or tasks [94]. It is plausible that muscle strength and endurance may be better predicted by task-specific psychological assessments instead of generic questionnaires, as has been shown for other types of movement behaviour (e.g., lumbar range of motion) [95,96].

In summary, interpretation of the performance on back muscle strength and endurance tests of an individual patient with CLBP is challenging. Interpretation is confounded by the many different methods to assess back muscle strength and endurance [74,97,98], variation in the patients’ functional demands (e.g., physical job requirements), and the capacity of muscles outside the lumbar region (hip or thoracic extensor muscles) to contribute to test performance [74,99]. With respect to this latter point—some studies report that performance on the Biering–Sorensen test is determined by fatigue of the hip extensor muscles [100,101]. Since dysfunctions in back muscle strength and endurance are common in patients with CLBP, these aspects require assessment. Yet, test results need to be interpreted carefully, keeping in mind potential confounding. 

### 2.4. Potential for Exercise Therapy to Address Back Muscle Dysfunctions

The literature summarized above supports the justification for consideration of the changes in structure and function of the back muscles as a component of a multifactorial program for the management of back pain. Addressing muscle changes in CLBP may involve strategies to reduce excessive protection, often involving overactivation of the more superficial erector spinae muscles, while also improving the function and structure of the deeper muscles, including the multifidus. Assessments of many aspects are likely to be necessary to identify the range of features that are critical to address in LBP treatment. These include, but are not limited to, assessments of movements [102], posture [103], psychological factors [94], and pain characteristics [10] to identify the specific aspects that need attention.

Specific exercises can improve the impaired back muscle functions that are targeted during treatment [104,105,106]. Although many studies have investigated muscle endurance and strength outcomes, there is also evidence that specific sensorimotor control training can change muscle recruitment of the back muscles [107]. 

Evidence of the impact of exercise for structural changes in back muscles is incomplete. Some evidence confirms the capacity of exercise to restore muscle size [108], and muscle fibrosis can be reduced by physical exercise in animals [109]. A recent systematic review concluded that the very limited evidence that is available suggests that fatty infiltration in back muscles might not be reversible with exercise therapy [110]. Interpretation of these data is not straightforward; the exercise programs used in some of the few available studies may have been too short and used insufficient loads to achieve structural changes [110], and whether the affected muscles were actually recruited during the training tasks was not addressed. Restoring fatty and fibrotic changes in muscle structure would likely require resistance training and be preceded by exercise to ensure adequate engagement of the affected muscles during the training tasks. Failure of a 16-week (3x/week; 6–10 RM) program of resistance training to reduce fatty infiltration at the lower lumbar spine (L5-S1) might relate to failure to engage these muscle areas in the training task [111]. In chronic LBP, it has also been shown that low-load motor control training alone is insufficient to restore muscle CSA [112], but combining it with controlled progressive overload training after low-load training can promote hypertrophy in the multifidus and reduce pain and disability [108]. This finding is supported by a more recent systematic review [113]. Considering the reduced proportion of type I muscle fibres in a subgroup of patients, endurance training may also be necessary. It is plausible to speculate that training for chronic LBP should initially focus on activation patterns tailored to individual adaptations, followed by resistance training for strength and endurance.

In conclusion, there is clear evidence that specific exercises can improve back muscle strength and endurance, and some studies also show that specific sensorimotor control training can change back muscle recruitment. The picture is less clear regarding the impact of exercise therapy on muscle structure, which may partly be explained by methodological limitations of the current literature. Exercise programmes of longer duration that initially focus on adequate muscle recruitment strategies followed by resistance training may be necessary to achieve changes in muscle structure.

### 2.5. Changes in Back Muscle Function and Clinical Parameters

Given the observed dysfunctions in patients with CLBP, lumbar back muscles are often targeted during exercise programmes. Because these exercise programmes typically lead to improvements in the targeted muscle-related (e.g., strength) and clinical (e.g., pain and disability) parameters [105,106,114], it is tempting to hypothesise that there is a causal relationship between these two. Although plausible, it remains unclear whether improvements in clinical parameters are contingent upon changes in lumbar back muscle function [115,116,117,118,119]. For example, Wong et al. (2014) concluded in their systematic review that the relationship between changes in multifidus (function) and clinical improvements are uncertain [115], although it must be acknowledged that many of the included studies used measures that lacked the capacity to evaluate the activation of the deep portion of the muscle. A systematic review by Steiger et al. (2012) showed that improvements in trunk extension strength were not associated with reductions in pain intensity and disability [116]. Again, the issue might be the lack of specificity of measures, as when the analysis is restricted to studies that evaluated lumbar extensor strength in isolation, positive correlations were found [120]. 

Various limitations of clinical studies require consideration when interpreting their results. First, clinical trials often do not consider the heterogeneity of the CLBP population. There is mounting evidence that patients with nociplastic pain (i.e., pain related to abnormal processing of nociceptive information [121]) or unhelpful beliefs (e.g., fear of movement) do not respond well to specific exercise therapy, such as muscle strengthening or motor control training [122,123,124,125]. Although these patients might achieve improvements in muscle function, this is unlikely to translate to clinical improvements [10]. 

Second, clinical trials typically assess movement behaviour (e.g., kinematics or muscle activity) in a generic manner, irrespective of patient presentation. If the movements and muscle-function parameters relevant for the individual patient are not considered, the relationships between changes in muscle function and clinical parameters are less likely to be observed [119,126]. In this respect, a systematic review by Wernli et al. (2020) showed that relationships between changes in movement behaviour and pain or disability in patients with LBP were only found in 31% of the comparisons in clinical trials [127]. In contrast, a different systematic review including only single-case designs—using more individualised measures—reported such relationships in 72% of the comparisons [126]. Although most studies have only assessed kinematic parameters (e.g., ROM), the few available case-studies assessing changes in back muscle activity in an individual manner also found relationships with clinical improvements [117,126]. An individualised approach may potentially lead to new insights regarding the relationships with clinical improvements. 

In summary, there remains uncertainty whether changes in back muscle function after an exercise program are causally related to clinical improvements. Evaluating the impact of treatment by using assessments tailored to the individual patient is worthy of investigation and likely to provide a more promising investigation of the question.

## 3. Framework for an Individualised Approach to Back Muscle Training in Patients with CLBP

**Figure 2:**
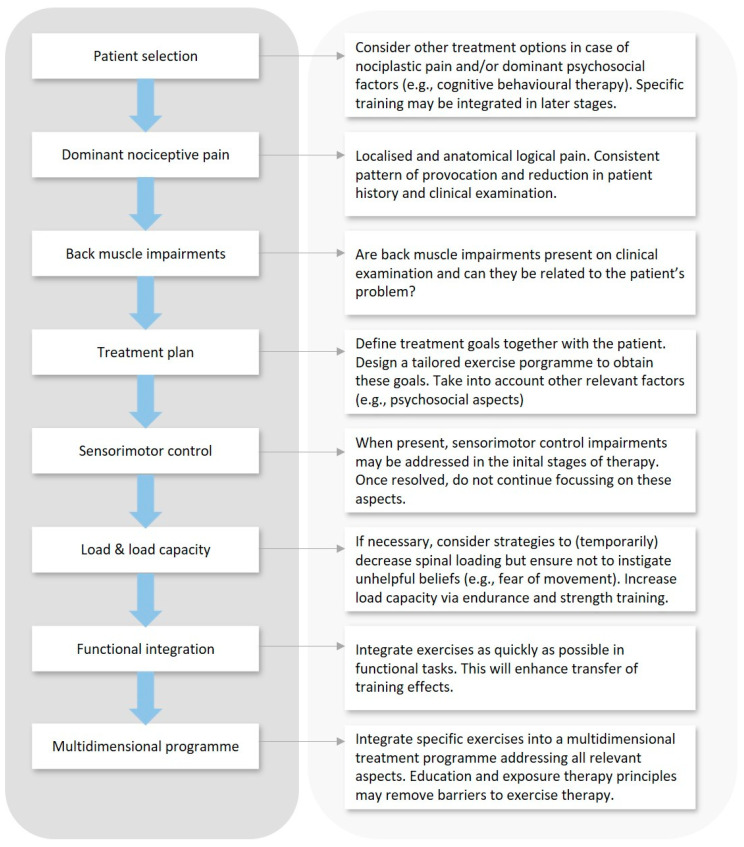
Overview of integrated framework for an individualised approach to back muscle training in patients with CLBP.

This section provides a framework for consideration of how back muscle training might be included in a comprehensive management plan for individuals with CLBP. An overview is provided in Figure 2.

### 3.1. Take the Heterogeneity of the CLBP Population into Account

Although exercise therapy is effective in reducing pain and disability in patients with CLBP, effect sizes are modest at best and not all patients respond well to specific exercises [114,128]. Moreover, systematic reviews typically show that one type of exercise therapy is not superior to another [114,128]. An important limitation of many clinical trials is that they provide exercise therapy in a non-individualised manner, failing to take into account the heterogeneity of the CLBP population [123]. A major challenge is thus to target patients who are likely to benefit from a particular treatment. 

There is increasing evidence that specific exercise therapy—i.e., sensorimotor control, muscle endurance and strength training—that focuses on changing how a patient uses their body and loads the spine is less effective for patients with strong unhelpful beliefs (e.g., fear of movement) or clear nociplastic pain characteristics [122,123,124,125,129,130] than for those with nociceptive pain (see below) [10]. For the former type of patients, other treatments such as cognitive behavioural therapy (e.g., exposure therapy in vivo to tackle avoidance behaviour) or more general exercises (e.g., aerobic activities) may be recommended [94,124,125,131,132,133]. In that case, encouraging a patient to get back to function despite their pain and regardless of how they move might be most critical (although avoidance behaviour should be addressed). While it could eventually be useful to integrate more specific exercises into these programmes (e.g., to address deconditioning), it is unlikely that this would be an effective target in the initial stages of the therapy. 

Specific exercises targeting the back muscles are probably more effective for patients with CLBP of predominantly nociceptive origin [122]. These patients have more localised pain with relatively clear patterns of provocation and reduction with specific postures and movements [134,135]. The premise is that suboptimal loading of spinal structures can be a cause of ongoing nociceptive input in many of these patients and a mechanism for the persistence of their CLBP. For individuals where this is related to postures and movements that involve activation of back extensors, addressing back muscle dysfunctions through specific exercise therapy has the potential to impact pain and disability secondary to optimised spinal loading [10]. This remains hypothetical and the exact mechanisms via which specific exercise therapy works are still largely unknown and are likely to be multifactorial [136].

Of note, within the subgroup of patients with nociceptive CLBP there is a large heterogeneity in clinical presentation and in back muscle dysfunctions. For instance, whether back muscles have high or low activity depends on clinical features of back pain, such as whether their pain is provoked by sitting in lumbar extension or flexion [103]. It would be expected that training to enhance back muscle structure and function would only be relevant for those who have clinical features that imply impaired structure and function and their relationship to pain provocation. Individualising exercise interventions based on a comprehensive patient history and clinical examination is paramount. A careful evaluation of the (painful) activities will guide treatment choices. For example, the modalities of back muscle endurance training can be different for patients who need to perform many repetitive flexion movements versus those who need to be able to maintain prolonged static semi-flex positions. In other words, effects of training are likely to be larger if treatments address features the patient lacks for participation in valued life activities. 

In summary, a critical first step in designing an intervention that includes consideration of the back muscles is to critically judge the potential pain mechanisms that might explain a patient’s pain. If nociceptive pain mechanisms are expected, then careful consideration of the patient’s presenting movement, posture, and back muscle structure would be relevant. 

### 3.2. Balancing Load and Load Capacity

In order to balance spinal loading and load capacity, patients with CLBP are often given advice and exercises that aim to protect the back and reduce spinal loading [137]. For example, they are taught to avoid sitting or lifting with a bent back. Although strategies to reduce spinal loading may be appropriate, it is critical that this does not lead to unhelpful beliefs such as ‘my back is fragile and needs protection’. These types of messages from health care practitioners are an important way for patients with LBP to acquire such unhelpful beliefs [137,138]. 

As there is evidence that many patients with CLBP have reduced back muscle strength and endurance [74,97] and that changes in muscle structure might require loading to be changed [108], therapists should aim to train with loads sufficient to induce strength and endurance improvement. Although load is unlikely to be harmful, it might be painful. Teaching patients to modify their movement prior to loading might be required. Increasing the load capacity is essential for functional reintegration, as many daily life, leisure and job-related activities require repetitive or prolonged muscle contractions. A careful analysis of these requirements can guide decision making during therapy. 

It is important to be aware that higher-load exercises might provoke transient back pain, and this is an important reason for patients to stop exercising [139,140]. Performance during strength exercises could also be impaired when patients expect them to be painful [93]. Pain education prior to participation in a (high-load) exercise programme may be helpful to remove barriers that may negatively affect adherence [141].

### 3.3. Sensorimotor Control Training

Earlier observations of structural and functional changes in the (deep) multifidus led to the development of specific sensorimotor control exercises targeting this muscle [37]. During these exercises, patients cognitively activate the (deep) lumbar multifidus independently from other back muscles [107]. It has been shown that changes in motor coordination could be reversed by these specific exercises, while this was not the case for simple back extensions activating all back muscles in a non-specific way [107]. Consequently, specific sensorimotor training of the multifidus has been advocated for patients with CLBP [12]. Although some have questioned whether such a specific approach is necessary because specific sensorimotor control exercises are not superior to general exercises to reduce pain and disability in patients with CLBP [142], other data suggest that this type of training might be more successful than general exercise when applied to patients with a consistent relationship between movements and pain (i.e., nociceptive type pain) [122]. This requires further investigation. 

It is logical that an exercise programme should target multiple components (e.g., sensorimotor control and muscle strength) and multiple muscles when appropriate. When sensorimotor control impairments are present, it may be recommended to integrate selective activation exercises of the multifidus in the initial stages of therapy. This can restore muscle activation patterns at the lower lumbar spine [107], which in turn might help to engage more caudal regions of the back extensor muscles during fatiguing exercises. This is highly relevant, because patients with CLBP activate more cranial regions of the back extensor muscles during these tasks [61,62,63,64], resulting in earlier fatigue [63] and increased pain [59,65]. However, whether sensorimotor control exercises can affect spatial distribution of back muscle activity is unknown. 

Once adequate sensorimotor control of the multifidus is achieved during selective activation exercises, it is unlikely to be necessary to continue to focus on this aspect and to transition to more functional training. Changes in muscle activation patterns obtained during selective training are likely to transfer to other activities [107]. Motor learning also needs to progress from an initial cognitive stage to more autonomous stages. A concern is that some individuals might become hypervigilant about the movements of their lower back as this may lead to unwanted protective movement behaviour [143].

### 3.4. Directed to Functional Integration

Analytical (non-functional) back muscle exercises can be useful when motor coordination is impaired or when the load capacity of the spine is low. In the last condition, analytical machine-based resistance exercises can be useful to create controlled overload in safe conditions in function of strength or endurance training [106]. However, training and rehabilitation should always be function-oriented. Therefore, we should, not hastily, but as soon as possible, start exercising in function of daily load and activities. In other words, training should integrate exercises into functional activities that are relevant for the individual patient and align exercise modalities with patient needs, as this allows for a better transfer of training effects.

Functional integration requires detailed assessment of the specific needs of a patient. This includes the functional evaluation of painful or frequently performed activities and adopted postures, with specific attention to habitual movement behaviour (for detailed description see [144]). The conclusions of this analysis should be translated to the choice of exercise modalities to individualise treatment. Examples include adaptation of movement speed, choice of functional positions, introduction of functional arm or leg movements, emphasis on static postures or dynamic movements, increased number of repetitions or introduction of dual cognitive tasks.

### 3.5. Integration of Back Muscle Training into a Multidimensional Treatment Plan

Although interventions that are limited to back muscle exercises have been shown to improve pain and disability [106], it is unlikely that this is ideal and that integration into a multidimensional treatment plan is likely to be more successful. There are multiple dimensions to consider.

Other physical aspects contributing to a patient’s problem should be considered. This might include training of other muscle groups, proprioceptive and movement coordination exercises, improving general physical fitness, and changing 24 h movement behaviour. Obtaining long-term behavioural change is challenging [145], but small adaptations such as movement breaks to interrupt static postures might already be useful [146]. 

Patients with nociceptive CLBP typically do not have a generalised fear of movement, but they might be afraid of certain activities [94,95]. When introducing functional back muscle exercises, such as lifting loads with a bent back, some patients might be afraid to perform them because they perceive these tasks as harmful [95]. Although pain education may be useful, it is often not sufficient to tackle avoidance behaviour [147,148], so principles rooted in exposure therapy may be necessary to address potential avoidance behaviour [94,131]. By letting patients experience that the expected catastrophe (‘My back will snap during lifting with a bent back’) does not occur, their expectation will be violated and they can learn that these activities are safe to perform [149,150]. This will increase confidence in their ability to perform these activities and it will extinguish avoidance behaviour [149], which in turn will decrease disability. A recent randomised clinical trial showed that this approach is superior to general exercise therapy for reducing pain and disability in patients with chronic spinal pain [151].

It is also likely that many other elements require consideration that will differ between individuals. This might include consideration of sleep hygiene, stress management, diet, an many other aspects of an individual’s lifestyle that can relate to pain [152,153]. Patients require a detailed assessment to guide individualised training.

### 3.6. Critical Appraisal

Although our framework for an individualised approach to back muscle training in patients with CLBP is based on the best available evidence, there remains uncertainty regarding various aspects that require clarification in future research. For example, the optimal exercise modalities to achieve changes in muscle function and structure are not always clear, and the relationships between these changes and clinical improvements require further investigation. Moreover, selecting patients that will benefit from a particular treatment is challenging given the heterogeneity of the CLBP population. Even within the subgroup of patients that is more likely to respond well to specific exercise therapy (i.e., those with dominant nociceptive pain characteristics), large variability in muscle function impairments is present. Careful analysis of muscle function is thus essential, yet not straightforward, especially in clinical settings where specialized equipment (e.g., electromyography) is often not available. Despite these limitations, the currently proposed framework provides clinicians with guidance on how to implement specific exercise tailored to the individual with CLBP.

### 3.7. Summary

Back muscle training is likely to be most effective if matched to the right patients and tailored to their needs and presentation. Exercise to target back muscle function and structure is likely to have its greatest impact on outcomes for patients with nociceptive CLBP. Within this heterogeneous group, back muscle dysfunction is not uniform and treatment plans would depend on findings of thorough patient history and clinical examination. Specific sensorimotor control exercises at the initial stages of an exercise programme may be useful to optimise muscle activation patterns, but progression should be made towards back muscle endurance and strength training. Gradually increasing the load is safe for most patients and should be encouraged, rather than risking hypervigilance and excessive protection of the back. Exercises should be integrated into functional movements relevant for the patient. Because higher load (functional) exercises may cause transient back pain, education and the application of exposure therapy principles may be necessary to ensure adequate engagement of the patient. For an optimal outcome, back muscle exercises should be incorporated into an individualised multidimensional treatment plan.

## 4. Conclusions

This paper provides an overview of changes in muscle structure and muscle function in patients with CLBP. The integrated framework proposed for back muscle training in this population is based on current knowledge. It is essential to acknowledge the large variability in back muscle dysfunctions between patients with CLBP, and to carefully interpret their role in the persistence of back problems for the individual person. A multidimensional approach to low back pain management is likely to be optimal.

## Figures and Tables

**Table 1:** Summary of changes in back muscle structure in patients with chronic low back pain.

Parameter	Summary of Changes in Patients with Chronic Low Back Pain
Cross-sectional area (CSA)	- Smaller bilateral CSA of multifidus; unclear for erector spinae and other back muscles.
Fatty infiltration	- Increased fatty infiltration in multifidus and to a lesser extent in erector spinae. - More fatty infiltration in continuous CLBP compared to noncontinuous CLBP.
Muscle fibre type	- Inconsistent results, but potentially dependent on LBP severity. Increased type II fibre proportion in patients scheduled for spinal surgery; no differences with pain-free persons in mild disabling CLBP.

**Table 2:** Summary of changes in selected back muscle functions in patients with chronic low back pain.

Parameter	Summary of Changes in Patients with Chronic Low Back Pain
Sensorimotor control	- Reaction times of erector spinae to predictable and unpredictable perturbations are inconsistent. When changes in erector spinae reaction time are found, they are typically delayed. - Increased activity of erector spinae during functional tasks, especially in patients with unhelpful beliefs. - Alterations in the motor cortex representation of the back muscles are present. - Patients with LBP weight down afferent proprioceptive information from back muscles during postural control tasks.
Spatial distribution	- Patients with CLBP activate more cranially located regions of back extensors during fatiguing tasks. - Unclear whether differences in spatiotemporal changes are present in CLBP. Spatiotemporal changes seem to vary depending on the task and the individual.
Muscle strength and endurance	- Decreased in CLBP, but strong inter-individual variability.

## Data Availability

Not applicable.
